# Treatment and Imaging Modalities of Giant Coronary Aneurysms Resulting from Kawasaki Disease and Presenting as Acute Inferior Wall Myocardial Infarction

**DOI:** 10.1155/2021/8878358

**Published:** 2021-01-16

**Authors:** Mahmood Abu Akel, Yaron M. Hellman, Shtiwi Sawaed, Erez Sharoni, Amnon Eitan, Moshe Y. Flugelman

**Affiliations:** ^1^Department of Cardiovascular Medicine, Lady Davis Carmel Medical Center and Rappaport Faculty of Medicine, Technion Israel Institute of Technology, Haifa, Israel; ^2^Department of Cardiothoracic Surgery, Lady Davis Carmel Medical Center and Rappaport Faculty of Medicine, Technion Israel Institute of Technology, Haifa, Israel

## Abstract

Giant coronary aneurysms are late sequelae of Kawasaki disease (KD). We describe a 53-year-old patient who presented with acute myocardial infarction and proximal aneurysms of all three coronary arteries. Coronary angiography demonstrated the aneurysms, but CT angiography allowed accurate assessment of the real dimensions of the aneurysms and making the decision on the preferred method of revascularization. The patient underwent coronary bypass surgery and is asymptomatic at follow-up.

## 1. Introduction

Kawasaki disease (KD) is an acute multisystem vasculitis of unknown etiology, which predominantly affects infants and young children. Fifteen to twenty-five percent of untreated patients with acute KD have coronary aneurysms or ectasia [1, 2]. Symptomatic coronary heart disease (CAD) develops in 5% of patients during long-term follow-up [3, 4]. Symptomatic CAD is due to narrowing or thrombosis of the coronary arteries secondary to the coronary aneurysms [5–7].

We present a 53-year-old man who presented with myocardial infarction and was found to have giant coronary aneurysms, probably due to KD. We discuss clinical management and review the relevant imaging modalities and potential therapies.

## 2. Case Presentation

**Figure 1:**
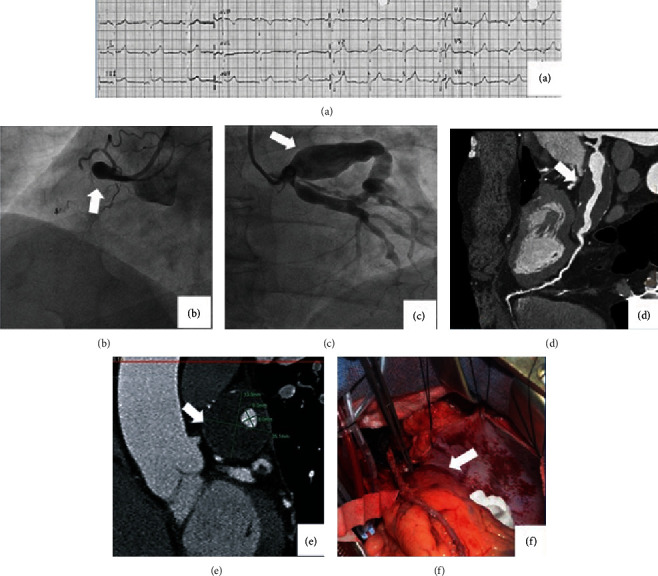
(a) Twelve-lead ECG on admission. ST segment elevation in leads II, III, and AVF is evident. Acute ST elevation inferior wall infarction was confirmed by troponin T dynamics over the following week. (b) Coronary angiography showing a large coronary aneurysm involving the proximal right coronary artery (white arrow) with complete occlusion of the artery. (c) Coronary angiography showing large coronary aneurysms involving the left anterior descending (black arrow) and circumflex (white arrow) coronary arteries. (d) CT angiography showing the gigantic proximal left anterior descending coronary artery aneurysm (white arrow). (e) Actual measurements of the left anterior descending coronary artery (LAD) aneurysm (white arrow): the maximal outer size of the LAD aneurysm is 35 × 33 mm, and the size of the lumen of the LAD is 9 × 11 mm. (f) A view from the operation showing the large aneurysm involving the left anterior descending artery (forceps tip and white arrow).

A 53-year-old man presented to the emergency department with chest pain that started on the day of admission. He was a smoker, with dyslipidemia treated by atorvastatin 20 mg, with no family history of cardiac disease or significant pediatric history. He was in good general condition and had no signs of heart failure. His ECG showed sinus rhythm and ST segment elevation in the inferior leads (Figure 1(a)).

The patient underwent emergency coronary angiography. The right coronary artery (RCA) was totally occluded with an aneurysm involving the ostium (Figure 1(b)). Filling of the distal RCA from the left coronary system was observed. Giant aneurysms in the proximal portions of the left anterior descending (LAD) and left circumflex coronaries (Figure 1(c)) were demonstrated. A second marginal branch was demonstrated with slow flow and filling defects.

An attempt to pass a guide wire to the proximal RCA through the ostial aneurysm failed, and the patient was transferred to the coronary care unit and treated with aspirin, clopidogrel, fractionated heparin, and morphine.

Echocardiography showed good left ventricular contraction with an estimated ejection fraction of 55% and wall motion abnormalities in the inferior and posterior walls. Peak troponin T was 1027 ng/l on the second day of hospitalization and decreased to 226 ng/l on the 7^th^ day of hospitalization (normal < 13 ng/l). The angiographic findings were attributed to KD based on the proximal location and size of the aneurysms and the lack of another explanation for these findings.

A cardiac computed tomography was performed, which revealed a giant thrombotic nonocclusive aneurysm in the proximal LAD with calcifications of the margins of the aneurysm (Figure 1(d)); the maximal outer size of the LAD aneurysm is 35 × 33 mm, and the size of the lumen of the LAD is 9 × 11 mm. Moderate aneurysmal dilatation of the proximal left circumflex and an ostial thrombotic aneurysm of the right coronary artery were observed.

One week after admission, the patient underwent coronary bypass surgery. The giant aneurysm of the proximal LAD (Figure 1(e)) was identified by the surgeons, and the left internal mammary artery was connected to the distal LAD. The right internal mammary artery was connected to the first marginal branch, and a saphenous vein graft was connected to the distal RCA.

Recovery from surgery was unremarkable, and a graded exercise test performed 3 months after surgery showed good aerobic capacity with no complaints or ST segment deviations.

## 3. Discussion

We describe a patient with an unusual cause of acute myocardial infarction at the fifth decade of life. The most probable etiology is thrombosis of coronary aneurysms, which are a late sequel of KD. Only a few reports have documented patients with giant aneurysms presenting for the first time in the fifth decade of life [7, 8]. Although diagnostic criteria have not been established for KD at the chronic phase of the disease, our probable diagnosis is based on the proximity and size of the coronary aneurysms and lack of signs of other syndromes that could explain coronary aneurysms, such as Behcet's disease [9]. In a previous report, we described acute coronary syndrome in a patient with KD, which was diagnosed retrospectively based on childhood medical records [10]. Since KD in childhood can be misdiagnosed or ignored, diagnosis during late phases of KD can be reached only with high probability based on anatomic findings and lack of signs of other relevant syndromes.

Our case report highlights several important issues. (a) KD can present late in life with no relevant childhood history. Although this is a rare presentation, it should be kept in mind in both young and older patients presenting with coronary aneurysms. (b) Combining different imaging modalities was important for better understanding of the anatomy and pathophysiology. We used coronary angiography and cardiac computed tomography and showed the exact size of the aneurysms and the healthier parts of the coronary tree that were suitable for bypass grafting. The use of multiple modalities shed light on the pathology of aneurysms in Kawasaki disease [11]. As shown in Figure 1(d), the lumen of the LAD is filled with contrast medium, while the area between the patent LAD lumen and the outer borders of the aneurysm is filled by “greyish” material that can be either organized thrombus or myofibroblastic proliferation and the resulting extracellular matrix [11]. Calcifications are also part of the pathophysiology of chronic Kawasaki disease affecting the vasculature [11].

Percutaneous interventions, bypass surgery, and medical therapy have been prescribed for KD presenting with acute coronary syndrome [6, 12–14]. In our patient, we decided jointly with our surgeons that the best therapeutic modality is coronary bypass surgery, based on the assumption that the thrombosed proximal coronary aneurysms can further limit coronary flow and cause additional myocardial injury and infarction. The size of the infarct was limited in our patient due to filling of the distal RCA from collaterals from the left system.

The relevance of our case report is highlighted by the potential increase of patients with Kawasaki disease-like syndrome during the COVID-19 pandemic [15–17].

## Data Availability

The data underlying this article will be shared on reasonable request to the corresponding author.
